# Editorial: Immune-related mechanisms in apicomplexan and trypanosomatid parasites

**DOI:** 10.3389/fimmu.2026.1965502

**Published:** 2026-08-25

**Authors:** David Arranz-Solís, Debanjan Mukhopadhyay

**Affiliations:** 1 SALUVET Group, Animal Health Department, Faculty of Veterinary Sciences, Complutense University of Madrid, Madrid, Spain; 2 Institute of Health Sciences, Presidency University, Kolkata, India

**Keywords:** host-parasite interaction, immune evasion, immune regulation, *Leishmania*, *Plasmodium*, *Toxoplasma gondii*, *Trypanosoma*

Host–parasite interactions are central to the outcome of protozoan infections. Intracellular parasites must establish a replicative niche while confronting immune mechanisms that restrict invasion, intracellular growth, dissemination and persistence. The disease pathology is not merely driven by the pathogen, but also by dynamic host-immune reprogramming that defines disease progression, persistence, and resolution. The survival of parasites inside host cells is not simply a consequence of limited immunity. It can result from the parasite’s ability to evade or withstand host defense mechanisms while simultaneously exploiting cellular stress, metabolic, and regulatory pathways. Conversely, protective immunity is not necessarily equivalent to excessive inflammation. Effective host defense appears to be dependent on achieving a carefully controlled balance between antimicrobial activity and preservation of host tissue homeostasis. This balance is particularly complex in apicomplexan and trypanosomatid parasites, which have evolved diverse strategies to manipulate host signalling, cellular metabolism and immune effector mechanisms. Understanding this dynamic interplay is therefore essential not only for explaining differences in virulence and disease progression, but also to identify targets for vaccines, diagnostics and therapeutic intervention. The Research Topic “Immune-Related Mechanisms in Apicomplexan and Trypanosomatid Parasites” was conceived to bring together recent advances in these areas, with particular emphasis on *Toxoplasma gondii* and *Leishmania* spp., while encompassing a broader range of medically and veterinary important parasites. The resulting Research Topic comprises 11 contributions, including nine original research articles and two reviews, covering Toxoplasma gondii, *Neospora caninum*, *Plasmodium* spp., *Babesia*, *Leishmania* spp., and *Trypanosoma cruzi*. Together, these studies illustrate the breadth of current approaches to understanding parasite–host interactions, from molecular mechanisms and cellular responses to *in vivo* pathogenesis and translational opportunities.

Several contributions emphasize the importance of innate immunity and the cellular context in determining infection outcome. Hanke et al. examined the very early responses of human and bovine monocytes and macrophages to *T. gondii* tachyzoites, showing rapid cellular activation and cytokine production, while extracellular-trap formation was comparatively uncommon during the first hours of exposure. In another *Toxoplasma* study, Ungogo et al. investigated interferon-stimulated mechanisms shared between humans and pigs. Using heterologous expression approaches, they identified IRF1 and IDO1 as conserved anti-*Toxoplasma* effectors and showed that human IRF1 could restore parasite control in IRF1-deficient porcine cells, providing mechanistic support for the use of pigs as a large-animal model for human toxoplasmosis.

The interaction between parasite virulence and host immunity is further illustrated by the works on *Neospora caninum* and *Trypanosoma cruzi*. Amieva et al. used CRISPR/Cas9-mediated deletion of the rhoptry protein NcROP2 to demonstrate that this parasite factor contributes to virulence in a pregnant mouse model. Loss of NcROP2 reduced parasite burden and clinical disease, enhanced interferon-stimulated responses in bovine macrophages and promoted transcriptional changes associated with stress and bradyzoite differentiation, linking parasite developmental plasticity with immune evasion. Valencia Ayala et al., in contrast, compared *T. cruzi* strains with distinct virulence phenotypes and found that enhanced early expression of antioxidant enzymes was associated with parasite persistence and virulence. Upregulated expression of antioxidant genes in the parasite contributes to lowering nitric oxide and reactive oxygen species generation in macrophages *in vitro* and increased parasite load *in vivo*. Their findings highlight antioxidant defenses as an important interface between parasite adaptation to host-derived oxidative stress and disease severity, and suggest potential biomarkers or therapeutic targets.

The *Leishmania* contributions provide several complementary perspectives on immune regulation and parasite adaptation. In canine visceral leishmaniasis, de Souza et al. characterized splenic macrophage phenotypes and identified simultaneous M1- and M2-associated features, together with markers of cellular exhaustion, in relation to parasite burden and splenic pathology. NOS2-positive macrophages reflect a nitric-oxide-associated antimicrobial response, whereas arginase-1-positive cells provide a metabolic environment favorable to parasite persistence. Importantly, higher parasite loads were associated with a lower NOS2/arginase-1 ratio, while CD206 and PD-L1 expression were associated with disease-associated splenic changes. These findings reinforce the concept that macrophage responses cannot be reduced to a simple M1/M2 dichotomy. Souza dos-Santos and Guedes reviewed the role of γδ T cells in human and experimental leishmaniasis, stressing their context-dependent production of IFN-γ, IL-17 and IL-10 and the critical influence that the parasite species, disease form and experimental models have on the disease outcome. These cells can produce both inflammatory and regulatory cytokines and may occupy different anatomical compartments within lesions. Their persistence even after clinical cure suggests that immune memory and surveillance may continue long after manifestations of infection have disappeared. Lobo-Silva et al. complemented these immunological studies with longitudinal transcriptomics in a murine model of cutaneous leishmaniasis caused by *L. braziliensis*. Their analysis identified temporal changes in inflammatory, macrophage-associated and tissue-repair pathways, and conserved microRNAs and epigenetic regulators with potential relevance for host-directed interventions. Importantly, approximately 77% of inflammatory pathways identified in human cutaneous leishmaniasis were recapitulated in ulcerated mouse lesions. Yet many inflammatory pathways remained active when the lesions were clinically healed, reinstating the fact that clinical resolution may not match molecular resolution. Finally, Fonseca et al. demonstrated that increasing trypanothione reductase activity in *L. tarentolae* enhanced resistance to oxidative stress and substantially increased infectivity, providing functional evidence that parasite redox homeostasis can influence the ability to establish infection and supporting this enzyme as a potential therapeutic target.

The Research Topic also illustrates how immunity can extend beyond conventional responses to a single parasite. Zafar et al. investigated heterologous protection between the related blood parasites *Plasmodium berghei* and *Babesia rodhaini*. Prior non-lethal *Plasmodium* infection reduced subsequent *Babesia* parasitemia and tissue damage, while macrophage depletion abolished this protection. This identified a macrophage-driven innate response associated with IFN-γ, IL-12, TNF-α and reactive oxygen/nitrogen species, balanced by IL-10. Donassolo et al. addressed adaptive immunity from a translational perspective by characterizing naturally acquired antibodies against the *Plasmodium vivax* liver-stage protein Pv_LISP-2 in individuals from malaria-endemic regions of Brazil. The detection of IgM and predominantly cytophilic IgG1 and IgG3 responses supports further investigation of Pv_LISP-2 as a candidate antigen for liver-stage vaccine development, while also demonstrating the value of studying naturally acquired immunity in exposed populations.

Finally, Hammi et al. provide a broader conceptual framework through a review of the integrated stress response in apicomplexan and trypanosomatid parasites. Their synthesis highlights how parasite-intrinsic stress pathways intersect with host stress responses and contribute to adaptation to nutrient limitation, oxidative stress and other hostile conditions during infection and developmental transitions. Viewed alongside the experimental studies in this topic, this review reinforces a recurring theme: immune evasion is closely connected to parasite metabolic and stress-adaptation programmes rather than being governed by isolated immune-modulatory factors.

Taken together, the 11 contributions demonstrate the multifaceted nature of parasite–host interactions and the value of examining immunity across scales, from individual parasite proteins to immune-cell behavior, tissue responses and naturally acquired immunity. A particularly striking feature of the Research Topic is the convergence of several studies on the concept of balance: effective host defense must restrict parasite replication, whereas successful parasites adapt to oxidative, nutritional and inflammatory pressures and can redirect host pathways to favor persistence. The studies also demonstrate the importance of comparative approaches across parasite species, parasite strains and host species, as well as the integration of genetic manipulation, transcriptomics, immunophenotyping and *in vivo* models. These advances may ultimately help bridge mechanistic parasite immunology with the development of vaccines and host- or parasite-directed therapies. We hope that this Research Topic will stimulate further interdisciplinary work aimed at defining the mechanisms that determine whether immune responses result in parasite clearance, persistence or immunopathology. We sincerely thank all authors who contributed to the topic, the reviewers whose expertise and constructive comments helped strengthen the manuscripts, and the Frontiers editorial team for their support throughout the process.

